# Mixed method survey to assess the problems and propose solutions for implementation of CME/CPD in Sindh, Pakistan

**DOI:** 10.12669/pjms.35.2.243

**Published:** 2019

**Authors:** Nighat Shah, Marium Soomro, Mehjabeen Musharraf, Shiraz Shaikh

**Affiliations:** 1 *Nighat Shah, MBBS, MCPS, FCPS, MRCOG, MHPE, APPNA Institute of Public Health, Jinnah Sindh Medical University, Karachi, Pakistan*; 2 *Marium Soomro, BDS, MSPH, APPNA Institute of Public Health, Jinnah Sindh Medical University, Karachi, Pakistan*; 3 *Mehjabeen Musharraf, MSC, MSPH, APPNA Institute of Public Health, Jinnah Sindh Medical University, Karachi, Pakistan*; 4 *Shiraz Sheikh, MBBS, FCPS, APPNA Institute of Public Health, Jinnah Sindh Medical University, Karachi, Pakistan*

**Keywords:** Accreditation, CME/CPD, Recredentialling

## Abstract

**Objectives:**

To determine the type of CME/CPD sessions attended by doctors, identify the problems with implementation of CME/CPD program and propose possible solutions for successful implementation of CME/CPD Program.

**Methods:**

This was a concurrent triangulation mixed method study. Quantitative questionnaires were filled by faculty and physicians from different specialties. The qualitative component had both focus groups and in depth interviews conducted with different professional bodies (PMA), faculty JSMU, College of Family Physicians. This study was done in 2016-2017.

**Results:**

More than half of the participants (53.6%) reported that CPD/CME should be mandatory. Doctors who had graduated from outside Karachi were significantly less likely to report CPD/CME to be mandatory as compared to graduates from Karachi (p=0.004).Top three factors which influenced HCP’s to attend CPD/CME included career progression (65.1%), interest (54.7%) and knowledge gap (50.5%). The most common perceived barriers were lack of study leave, cost and work-life imbalance. The implementation issues expressed by different stakeholders included lack of doctor’s interest, busy clinical schedule and poor accessibility.

**Conclusion:**

Mostly medical practioners believe that CPD program is an important step toward enabling physicians to demonstrate their competency and professionalism to themselves and others. The PM&DC has to take practical steps to evaluate the cognitive, clinical, and humanistic attributes of certified doctors.

## INTRODUCTION

A quote from Maimonides’ *Daily Prayer of a Physician says*: “May there never develop in me the notion that my education is complete!”

In this context we inculcate the idea of lifelong learning; Continuous Medical Education (CME) and Continuous Professional Development (CPD) are essential components of lifelong learning. These are the cornerstones through which the medical practitioners update their knowledge skills and attitudes, focusing on social, communication, personnel and management skills.^1^

The basic medical degree enables a physician to practice medicine but lifelong medical practice cannot be dependent on that alone. Considering the speed with which medical science progresses, physicians must stay abreast with latest developments to provide quality care to their patients. At present, a *Medicinae Baccalaureus, Baccalaureus Chirurgiae (MBBS*) degree in Pakistan is life-long license for practicing medicine.^2^

It is indeed the need of the hour to provide evidence that we respond to emerging health needs through CME/CPD. It is the responsibility of academic institutions and accrediting bodies to set up CPD programs for recredentialling.^3^

Globally different professional bodies like American Board of Internal Medicine (ABIM), United Kingdom (UK), the General Medical Council (GMC) recognized the need of CMP/CPD. It was linked to revalidation of license to practice. Hence the process of recertification is mandatoryfor public accountability and professional obligations of self-regulation.^4^

In Pakistan the knowledge, skills and service delivery has very little check and balance and physicians practice norms are often not updated. The Physicians today are challenged not only by the diseases and their changing patterns, but on the socio-political front Pakistani media and courts are asserting their independence and enforcing accountability. External checks through stories in the country’s news media and petitions in court are fast becoming a norm. The present health system is facing difficulty in coping with growing pressures from the civil society. The cover stories of medical malpractice and negligence are routine on electronic, social and print media.^5^

In view of these external checks and new challenges, Pakistan Medical and Dental Council (PMDC) has made CME mandatory for doctors. Fifteen credit hours for general practioners and 30 credit hours for specialists but the opportunities for these are sparse and limited.^6^

The College of Physicians and Surgeons of Pakistan (CPSP) is conducting certifying courses but these programs occur on a small-scale, largely ad-hoc basis. As such the PM&DC must play a proactive role as a custodian of continuous medication education in Pakistan.^7^

The objectives of this study therefore were to determine the type of CPD’s attended by doctors in the last 12 months, identify the problems with implementation of CME/CPD program and propose possible solutions for successful implementation of CME/CPD Program.

## METHODS

It was a concurrent triangulation mixed-methods study.

### Quantitative methods

Quantitative data was collected through a self-administered structured questionnaire. The practicing physicians both males and females at Jinnah Sindh Medical University (JSMU), family physicians, and physicians from different fields filled the questionnaire survey.

Sample size was calculated by using statistical software “Open Epi”. Expecting a frequency of 50% for all types of CPD’s attended, at confidence level of 95% and bound on error of 5%, sample size came out to be 385. Informed consent was taken. The questionnaire used for quantitative data collection was a validated tool taken from GMC and Academy of Royal Medical colleges (open source) but was contextualized according to the local scenario and piloted at APPNA Institute of Public Health (AIPH). Expert opinion was taken for the face validity and content validity.

### Qualitative methods

Qualitative data was collected through in-depth interviews (IDI) and focus group discussions (FGDs) conducted by principal investigator (PI). Three IDIs were conducted with Vice Chancellor JSMU who also represents province of Sindh at PMDC, Dean AIPH(PhD in medical education) and Ex Federal DG Health.

Three FGDs were conducted with Head of Departments of Jinnah Sindh Medical University and affiliates, representative of Pakistan Medical Association and Family Physicians.

The qualitative data collection team included the moderator (PI), note taker and a support person to supervise the audio and videotaping. Moderator used semi-structured questions to elicit participant’s responses. The semi structured interview guide included the following four main questions:

1. What are your perceptions on CME /CPD?
2. How do you think CME/CPD effect the knowledge and practice of HCP?
3. What are the problems with implementation of CME/CPD?
4. What are the possible solutions for successful implementation?

The interview was piloted with one of the key stakeholder prior to implementation. The average duration of IDI and FGDs was 60 minutes. All interviews were conducted in English and Urdu (the national language of Pakistan) and took place over period of one year June 2016 to June 2017. Both the participants and the study member accompanied by note takers were alone during the interviews. Prior to each interview the participants were informed of the reason for the study and verbal and written informed consent was obtained.

### Plan of Analysis

### Quantitative Analysis

All the categorical variables including the characteristics of participants, their preference for CPD, common types of CPD attended by them, factors influencing them to attend CPDs and perceived barriers to attend CPD were summarized as frequencies and percentages. Differences in proportion of Health Care Professional’s (HCPs) reporting CPD/CME to be mandatory between age, gender, current position, current institution and graduating institution were analyzed using chi-square test. P-value of <0.05 was considered significant. Analysis was conducted on SPSS version 20.

### Qualitative Analysis

Qualitative analysis was conducted using thematic framework analysis. The transcription of data was simultaneously done after the IDI and FGD were conducted. All the interviews were translated to English. To maintain anonymity, participant names were not included on transcriptions. Two individual researchers separately analyzed all the transcripts, independently reading and identifying the responses to be listed down below the pre identified themes. Investigators met to review coding of the transcripts. One additional study member expert in qualitative methods independently reviewed the coding and categories; disagreements were reviewed and resolved by a third study member. Consensus was reached and the deductive analysis was done according to the defined coding system. The responses were later condensed into sub themes.

### Ethical considerations

The institutional review boards (IRB) from Jinnah Sindh Medical University, Karachi, JSMU/IRB/2016/-36 approved the study proposal.

## RESULTS

### Survey Findings

The mean age of the participants was 34.2 (SD=9.57). Females (55%) outnumbered the males (45%). The participants were almost uniformly distributed among different junior and senior positions and between public and private institutions (Table-I). More than half of the participants (53.6%) reported that CPD/CME should be mandatory.

**Table-I:** Descriptive Statistics of Study Participants (n=289).

	% (n)
***Age***	
Mean +-SD	34.2+-9.57
***Gender***	
Male	45% (130)
Female	55% (159)
***Current Position***	
Lecturer/Instructor	24.9% (72)
Intern/Postgraduate trainee	29.1% (84)
Medical Officer/Woman medical Officer	25.3% (73)
Assistant Professor and above/Consultant	20.8% (60)
***Current Institution***	
Jinnah Sindh Medical University	27.3% (79)
Dow University of Health Sciences	16.6% (48)
Aga Khan University	11.1% (32)
Liaquat University of Medical and Health Sciences	15.2% (44)
Other Private Institutions	29.8% (86)
***Graduating Institution***	
Medical Institution in Karachi	66.8% (193)
Medical Institutions from Sindh outside Karachi	33.2% (96)

No significant difference in HCP’s reporting CME/CPD to be mandatory was found in relation to age and gender. Although senior cadres of HCP’s reported higher frequencies of CPD/CME to be mandatory, the differences were found statistically insignificant (p=0.433). HCP’s who had graduated from outside Karachi were significantly less likely to report CPD/CME to be mandatory as compared to graduates from Karachi (p=0.004). The five most common CPD/ CME events attended by HCP’s in the last 12 months included conference (62.6%), training (50.2%), reading articles (50.2%), seminars (42.9%) and drug company event (41.2%). Top three factors which influenced HCP’s to attend CPD/CME (Fig.2) include career progression (65.1%), interest (54.7%) and knowledge gap (50.5%). Other less commonly reported factors included collecting CPD points, reflection on practice and part of annual appraisal. Majority considered CME/CPD to be a professional necessity and critical to improve medical practice. More commonly preferred methods of learning included problem solving (52.6%), group work (47.8%) and workshop (45%) while less commonly reported methods included lecture, self-reading, and online courses. The most common perceived barriers were lack of study leave, cost and work-life imbalance (Fig.3).

**Fig.1:**
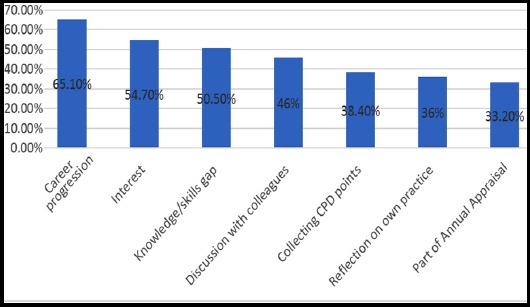
Predominant factors which influenced HCP’s to attend CPD/CME (n=289).

**Fig.2:**
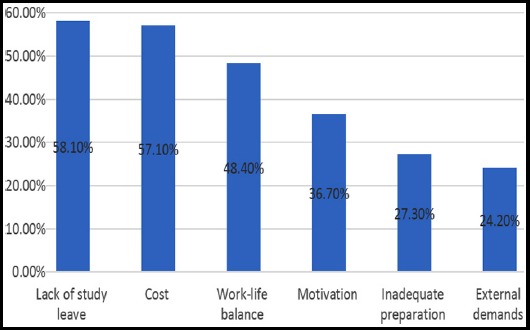
Perceived barriers to CPD Learning (n=289).

### Qualitative findings

The three pre-identified themes were perceptions about CME, Implementation Problems and Solutions for Implementation. Themes with subthemes are listed below.

1. ***Perception of CME***
  - Continuous up gradation
  - Need for all regardless of experience
  - Renewal of Information
  - Positive Intervention
2. ***Problems in implementation***
  - Personal Barrier (time/cost)
  - Accessibility
3. ***Solutions for implementation***
  - Introduction of e CME
  - Yearly schedule of CME
  - Controlling fake Certifications

### TYPE of CME/CPD

Participants consider that the continuous up gradation of knowledge in the field of medicine is necessary for practicing physicians. There are many well-known practitioners who are very experienced but they have no idea about modern research and knowledge.

Juniors usually take CME sessions. They also access new knowledge using the Internet and reading journals and get updated. But seniors usually don’t use technology. CME should be there for everyone at all levels. (JSMU Faculty).

One of the participants mentioned that CME and CPD through e-learning were relevant to renew information, and to deliver the services effectively.

The knowledge we get from books becomes obsolete after 5 – 10 years because new research and knowledge comes (PMA officials).

### Implementation problems

While talking about implementation issues, participants mentioned that medical professionals are themselves not willing to acquire new skills. Even if they attend the sessions, they are just interested in getting credit hours rather than learning. Also, due to the hectic clinical practice, doctors don’t get time for up gradation of skills. Other problem is of access, as only few academic centers offer CME, mostly in major cities. Despite implementation of several CME programmes in cities, doctors in many rural areas have little or no access to these courses. Although there is an established code to complete 30 hours of CME/5 years, only doctors in teaching hospitals comply with this, as it is not legally binding.

### Implementation solutions

Participants suggested the solutions for the effective implementation of CME. They said that there should be online CME sessions probably on cell phone, to resolve the issue of accessibility. In addition, a complete yearly schedule of all sessions should be made with the dates and venue mentioned so that physicians can mark their calendars accordingly. The smooth implementation of CME also requires check and balance mechanism to make sure that fake certificates are not made.

There should be programs available all year round so that people can avail them whenever they have the need, it should not be restricted to a particular time or month (medical educationist/PMDC).

## DISCUSSION

The results of this study show that the health care providers (HCP) are cognizant about the need for CPD/CME. Generally they agreed on both qualitative (all experts) and quantitative forums that it should be mandatory. It was largely seen that physicians from different specialties stressed the need for learning. However, 46.4% physicians who wished for voluntary CME were from rural remote areas. Similar findings were observed by Siddiqui et al.^8^ In this study however almost 100% in qualitative arm insisted for mandatory CME to improve patient outcomes. Studies in neighboring countries have also shown that mandatory and consistent CME is significantly more associated with improvement in the concepts and approach towards quality patient care.^9^

The responsibility that physicians have to continue to learn throughout their professional lives has been stressed by code of conduct of American Medical Association^10^ this same was stressed by Pakistan Medical Association FGD that physicians shall study and apply knowledge for betterment of patients and public.

### Professional development and attitude change

The CME/CPD’s that our physicians most frequently attended included conferences, trainings, and journals; PM&DC also in its notification for CME credit hours recognizes conferences and workshops by recognized institutes mostly in big cities for CME credit hours.^11^

The experts in qualitative arm were interested in professional development that caused practice change or change in attitude and behavior. They maintained that CME for collecting certificates and CME points should be discouraged and quality professional development should be enhanced for betterment of patients and community. This is in concordance with studies by SE Nissan^12^ and similarly Fox and Bennett emphasized adult learning through peer and experts for best practices.^13^

The main benefit of CPD as mentioned by Health professionals in the qualitative arm is inculcation of lifelong education for physician and patient benefit. In Canada, UK, and New Zealand the accrediting body and medical councils are implementing the competency based CPD for life-long learning culture.^14^,^15^

Mostly the physicians agreed that the system of CPD is lacking in monitoring and evaluation. Globally there is transparency and accountability through evaluations and standard setting of CPD to improve patient care.^16^

Usually the reasons stated for attending CME/CPD were career progression and covering gaps in the knowledge and practice. Similar factors for seeking CPD have been stressed by Mann and Gorden, as the health professionals recognized that they have to refresh and update their knowledge and skills to solve complex patient issues.^17^

### Challenges/Difficulties

The main difficulties highlighted in our study were time constraints leave from work place, and cost. In addition, as CME caters to the longest period of learning, the issues of making a credible, dynamic, reliable, cost effective and feasible CME/CPD which caters to the needs of profession.^18^

Establishing a widely accepted scheme of CPD that is sustainable and of the desirable quality is fraught with difficulty. In Pakistan there are more than 1,90,325 registered practitioners as per PM&DC in 2018. The increase per annum is 13%, having a support system to address their lifelong learning is challenging. In many studies similar trend has been seen as clinician often fight a losing battle to balance work, family and professional development challenges.^19^

In many countries in Southeast Asia, recertifiction has been linked to CME but it is difficult because of inappropriate CME programs and lack of motivation.^20^

Other issues, which were highlighted, were low quality programs, mostly information driven by pharmaceuticals. The health professionals were using old and obsolete information to manage new and difficult cases and there is an utmost need for appropriate CME to bring doctors and patients on the same platform.^21^ Therefore by and large the profession, patients and society are disappointed. This calls for urgent remediation by accrediting body.

### Solutions for Implementation

The experts in qualitative group supported that PMDC should have robust programs and systems with internal checks for revalidating. As the governing body for medical profession, PMDC has to urgently act to make the regular monitoring of doctors’ competence mandatory and to develop a program for continuous professional development. The solutions for implementation are straightforward and lie in overcoming the barriers (time, cost, accessibility) as per medical education experts interviewed in this study. This could easily be resolved if CME/CPD is electronically available as proposed in various studies.^22^ Or as a more smart option an app on smart phone as everyone now has cell phones. The data from research shows that e Learning is important source for CME. It is a rich resource which addresses multidimensional and complex information.^23^

The experts proposed that CPD has to be inculcated in system and accountability has to be conducted by accrediting body like PMDC. This should be mandatory for revalidation of licensure and fitness to practice. Some studies have suggested international or non-governmental organizations to monitor CPD. However our study showed that the physician expectations from PM&DC were high and that they maintained that PM&DC should play a proactive role in all aspects of CPD in order to achieve the desired results.^24^

### Strengths and Limitation

The strength of this study is that it deals with barriers/challenges and then the proposed solutions for implementation of CME/CPD, comprehensively through qualitative and quantitative methods. The recommendations regarding successful implementation of CME/CPD will be shared with the accrediting bodies of Pakistan.The limitation of this study was that some stakeholders were inaccessible, and sampling was not randomized but was convenient.

## CONCLUSION

The medical fraternity’s perceptions regarding preferable CME/CPD may differ but mostly they agreed to the importance of CME/CPD for improving patient outcomes. The CPD program is an important step toward enabling physicians to demonstrate their competency and professionalism to patients/society. The main barrier of time and cost could be overcome by easily available CME/CPD as an app on smart phone. The PM&DC as a mother organization has a mandate to ensure it. A collaborative effort by the industry, professional associations, regulators and accreditors may be instrumental in improving the processes and outcomes of medical education.

### Authors’ Contribution

**NS:** Conceived, designed, did data collection, statistical analysis, drafting, editing and review of manuscript.

**MS:** Literature search, data collection, writes up of discussion and reviewed the manuscript.

**MM:** Data collection, write up of methodology and discussion and reviewed the manuscript.

**SS**: Statistical analysis, write up of results and editing.
